# Behavioral Aspects of Phlebotomine Sand Flies Associated with a Case of Cutaneous Leishmaniasis in Atlántico, Northern Colombia

**DOI:** 10.4269/ajtmh.19-0245

**Published:** 2020-02-10

**Authors:** Erika Santamaría, Olga Lucía Cabrera, Catalina Marceló, Sergio Goenaga-Olaya, Ronald Maestre-Serrano

**Affiliations:** 1 Grupo de Entomología, Instituto Nacional de Salud, Bogotá, Colombia;; 2 Secretaría de Salud del Atlántico, Barranquilla, Colombia;; 3 Facultad de Ciencias de la Salud, Universidad Simón Bolívar, Barranquilla, Colombia

## Abstract

After the first autochthonous case of cutaneous leishmaniasis was reported in the Atlántico department in the Caribbean region of Colombia, entomological sampling was conducted in the specific areas where the infection might have occurred. CDC traps were installed inside and outside dwellings in the peri-urban and rural areas of a settlement in the municipality of Luruaco. Sampling was performed during the night with protected human bait, and phlebotomine sand flies were actively sampled from potential diurnal resting sites within dwellings. Ten species of the genus *Lutzomyia* were identified; *Lutzomyia evansi* was the dominant species (78%) in the rural and peri-urban areas as well as in the different sampled habitats, followed by *Lutzomyia panamensis* and *Lutzomyia gomezi*. There was a 100% household infestation by *Lu. evansi*, and its indoor mean abundance was 13.3 sand flies/CDC trap/night. The indoor mean abundance of *Lu. panamensis* and *Lu. gomezi* was only 0.9 and 0.8 sand flies/CDC trap/night, respectively. Female *Lu. evansi* were collected with protected human bait, mostly in the peridomestic area, with sustained activity during the night and a slight increase in the activity from 19:00 to 23:00 hours. Of the total sand flies captured in the diurnal resting sites, 73.1% were collected from the walls of bedrooms and corresponded to *Lu. evansi*, *Lutzomyia cayennensis cayennensis*, and *Lutzomyia trinidadensis*. Owing to their vectorial importance, the species on which entomological surveillance should be focused are *Lu. evansi*, *Lu. panamensis*, and *Lu. gomezi*. The biting and resting behavior reported in this study will help guide vector prevention and the control of leishmaniasis within the study area.

## INTRODUCTION

Leishmaniasis is a neglected infectious tropical disease caused by protozoan parasites of the *Leishmania* genus. The disease has different clinical manifestations ranging from cutaneous leishmaniasis (CL) with skin lesions to mucocutaneous leishmaniasis involving the mucous membrane to a lethal systemic form, visceral leishmaniasis (VL).^1^ Leishmaniasis is a crucial public health concern in Colombia. A total of 223,640 cases were reported between 2000 and 2016, with an estimated 12,000 cases per year since 2005^2^; however, the situation can be even more worrying, owing to a high number of underrecorded cases. The most common form is CL.

The Atlántico department in the Caribbean region of Colombia is considered a risk zone for vector-borne diseases. This area has ecological conditions that lead to the presence and proliferation of vectors both in rural and urban areas. Moreover, this department is the most economically developed area in the Caribbean region; hence, it receives immigrants from other regions of Colombia and from other countries where vector-borne diseases show endemic behavior.^3^

According to the Colombian National Epidemiological Surveillance System (SIVIGILA), until 2008, all Colombian departments other than San Andrés Islands and Atlántico reported cases of leishmaniasis. In July 2009, the health authorities of the Atlántico department warned about the possibility of a CL case in a 65-year-old man in the Santa Cruz settlement of Luruaco municipality (Secretaría de Salud del Atlántico, Epidemiology Reports, 2009, unpublished data). The man presented an atypical skin lesion on his chest, and the presence of amastigotes of *Leishmania* was confirmed by histopathological biopsy examination (Pathology Laboratory, National Institute of Health, Colombia, unpublished data). The affected person was mainly engaged in agricultural activities and stated that he had not traveled outside of the Atlántico department in the year before lesion appearance.

This was suspected to be the first autochthonous case of CL in the Atlántico department, and an entomological study was conducted in the specific areas where the infection might have occurred. This characterization was performed in 2009, 1 month after the case was confirmed, and the presence of the following ten species of the genus *Lutzomyia* was reported for the first time in the Atlántico department: *Lutzomyia evansi*, *Lutzomyia panamensis*, *Lutzomyia gomezi*, *Lutzomyia dubitans*, *Lutzomyia trinidadensis*, *Lutzomyia cayennensis cayennensis*, *Lutzomyia rangeliana*, *Lutzomyia punctigeniculata*, *Lutzomyia shannoni*, and *Lutzomyia camposi* (Maestre-Serrano et al., 2010, unpublished data).

The present study provides details on the entomological characterization performed in 2009, including the identification of habitats with a higher abundance of potential vectors of *Leishmania* spp. and the times of greatest biting activity and diurnal resting sites in habitats associated with the dwellings. Here, we aimed to define specific sites with a greater risk of human–vector contact and to increase the knowledge about the behavioral aspects of vectors that allow the recommendation of vector control measures for CL to be made.

## METHODS

### Study area.

**Figure 1.**
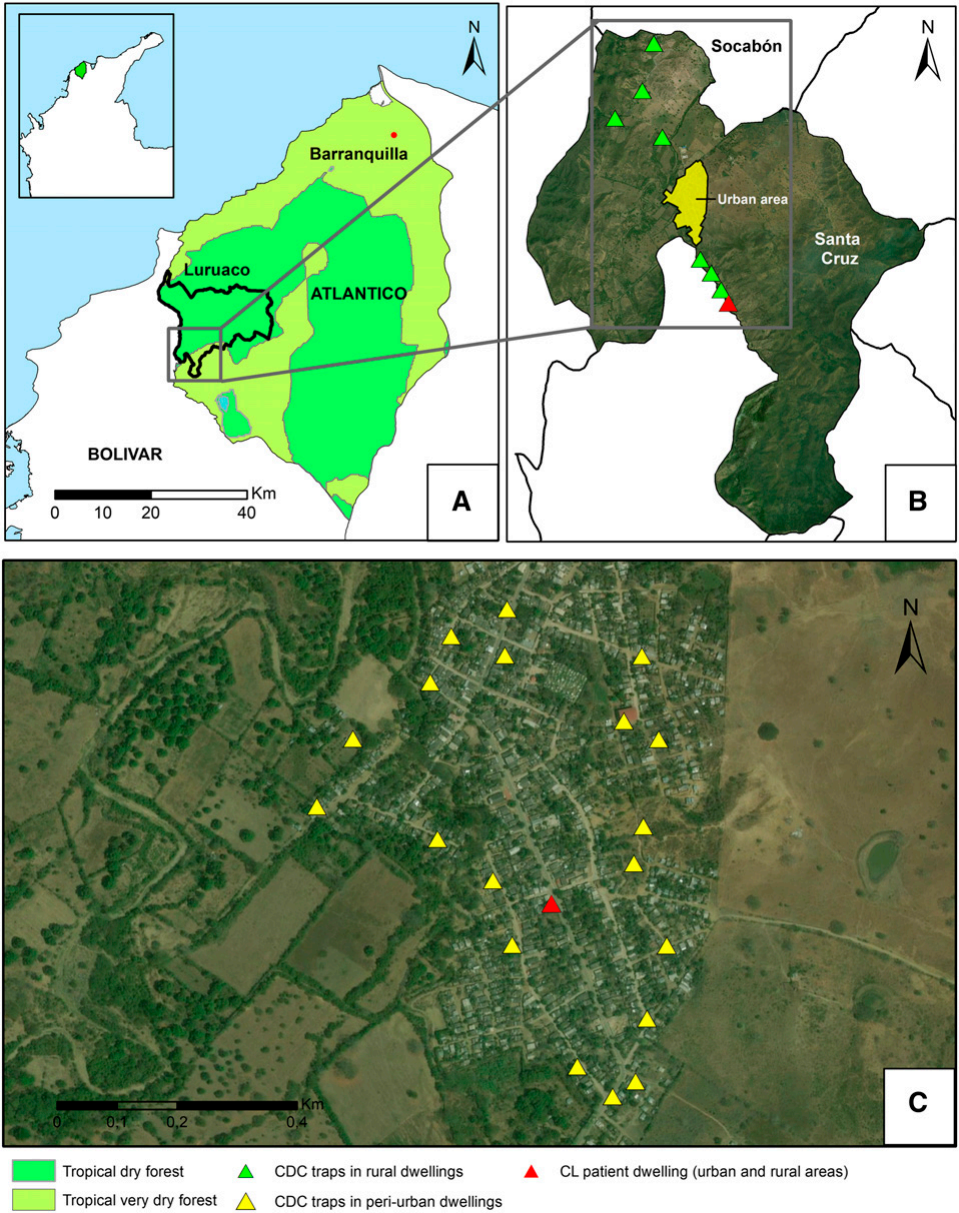
Study area. (**A**) Location of the Luruaco municipality within the Atlántico department. (**B**) Sampling area within of the Santa Cruz settlement and locations of the CDC traps in the rural area. (**C**) Details of the location of CDC traps in the peri-urban area.

This study was conducted in the Santa Cruz settlement (10°34′21″N; 75°12′47″W) of the Luruaco municipality located in the southwest of the Atlántico department. The settlement has an altitude of 31 m above the sea level and is situated 12 km from the Luruaco municipal seat and 67 km from the department capital (Figure 1A). Its average temperature is 28°C, relative humidity is 75%, and average annual precipitation is 800 mm^4^; according to the Holdridge life zones, the area is classified as a tropical dry forest. The area is under heavy livestock and agricultural pressure. The population livelihood is based on cattle ranching and agriculture, primarily sugar cane, millet, rice, and cassava cultivation.

### Phlebotomine sand fly sampling.

A descriptive study was performed in consideration of *Lutzomyia* spp. abundance per sampled area (rural and peri-urban areas) and habitats (indoors, peri-domestic areas, and outdoors).

### Indoors and peri-domestic areas.

In the peri-urban area of the Santa Cruz settlement, samples were collected from the dwelling of the patient with skin lesion. To select other sampling points, with the use of a settlement map, houses located on the periphery were numbered, and 19 of them were randomly sampled (Figure 1C).

In the rural area, two points were selected: 1) the rural area approximately 4 km from the urban area, located to the southeast of the settlement, where the CL patient had stayed overnight occasionally 2 months before the lesion onset and 2) the rural area in the opposite cardinal point, located to the northwest of the settlement urban area approximately 6 km on the road leading to the Socabón township. Four houses were selected at each point (Figure 1B).

Sampling was conducted using CDC light traps in the aforementioned points (20 dwellings in the peri-urban area and eight in the rural area). The traps were installed 1.5 m above the ground level from 18:00 to 6:00 hours in two types of habitats: indoor (defined as the occupied bedrooms of houses) and peri-domestic area (defined as the open areas of houses, including courtyards, yards, and animal sheds up to 15 m away). The houses had been continuously inhabited for at least 3 months before sampling.

### Outdoors.

In the rural area, forests were sampled using CDC traps (*n* = 4) at distances ranging from 100 to 400 m from dwellings. These forests had two arboreal strata and had coverage percentages between 50% and 70%. In the peri-urban area at distances between 50 and 100 m from the nearest dwelling, a habitat significantly impacted by human activity and defined as shrubland generally consisting of bushes (30%), pastures (30%), and other herbaceous plants (40%) was sampled using CDC traps (*n* = 8).

In addition, collections with Shannon trap were performed, and a greater abundance of phlebotomine sand flies was obtained using CDC traps outside the dwellings. A single sampling, from 18:00 to 21:00 hours, was performed in three locations: peridomestic and forest in the rural area, and shrubland in the peri-urban area.

### Phlebotomine sand fly human landing activity and resting sites in rural areas.

The human-landing activity of phlebotomine sand flies was established based on an observational study of *Lutzomyia* spp. abundance per hour performed from 18:00 to 6:00 hours in the indoors and peridomestic areas. The sampling method was catches by protected human bait. Four dwellings in the rural area were selected, where the highest abundance of phlebotomine sand flies was observed with CDC trap catches. Two volunteers, each one of them with 2-hour collection shifts, were in each habitat. Only the legs of the volunteers (from the knee to the ankle) were exposed, and the other body parts were fully covered. *Lutzomyia* females were caught using mouth aspirators as soon as they landed on the skin but before they bit. This procedure was replicated for two consecutive nights in each house, with the volunteer pairs rotating between habitats. All specimens captured within an hour were stored in plastic containers with plaster. The next morning, the phlebotomine sand flies were killed by freezing and preserved until their identification in the laboratory.

Regarding the search for phlebotomine sand flies in potential diurnal resting sites, rural area dwellings that were sampled with CDC traps (*n* = 8) were inspected indoors using a mouth aspirator and flashlight, specifically the bedroom walls, doors, and furniture. In the peridomestic area, the external walls, bathrooms, and shelters for domestic animals as well as tree trunks were inspected within a radius of 15 m. These samplings were performed from 8:00 to 11:00 hours. Each dwelling was inspected by three people within an average period of 30 minutes.

In all catching methods, the phlebotomine sand flies were cleared in the laboratory in 10% hot potassium hydroxide and transferred to saturated phenol.^5^ Species identification was made according to the classification adopted by Young and Duncan.^6^

### Data analysis.

To guarantee data quality, specific formats were designed for data collection. In the field, formats were checked in search of possible registry errors and missing data, and necessary corrections were made. The data were transferred to databases created in the Epi Info V. 3.5.4 program. The relative abundance of each species caught in the CDC traps, as well as resting phlebotomine sand flies collected by direct aspiration, is presented as percentages. For the three species with remarkable vectorial importance, abundance data per habitat are presented as the geometric mean of Williams (GM_w_) of the number of sand flies/CDC trap/night with 95% CIs. Considering that the data did not have a normal distribution, comparison was made through nonparametric statistical tests (Kruskal–Wallis test and Mann–Whitney U-test). The human-landing rates are presented as the GM_w_ of the number of females/person/hours, although just for the most abundant species, such as *Lu. evansi*.

## RESULTS

### Sampling with CDC traps.

The composition and relative abundances of the phlebotomine sand fly species caught using CDC traps are shown in Table 1. Ten species of the *Lutzomyia* genus were identified from 3,018 sand flies. *Lutzomyia evansi* was the predominant species (78%), both in the rural and peri-urban areas as well as in the three habitats sampled. *Lutzomyia panamensis* was the second most abundant species (12.5%), although it was found mainly in sampling sites outside houses. Other species with a low relative abundance, that is, < 5%, were *Lu. gomezi*, *Lu. dubitans*, *Lu. trinidadensis*, *Lu. c. cayennensis*, *Lu. rangeliana*, *Lu. punctigeniculata*, *Lu. shannoni*, and *Lu. camposi*. The mean abundance per habitat and sampling site for *Lu. evansi*, *Lu. panamensis*, and *Lu. gomezi* is presented in Table 2.

**Table 1:** Composition and relative abundance of *Lutzomyia* spp. caught with CDC traps in Santa Cruz settlement (Atlántico, Colombia).

Phlebotomine sand fly species	Rural area												Peri-urban area													
	Indoors				Peri-domestic area				Forest				Indoors				Peri-domestic area				Shrubland					
	*n* = 8				*n* = 8				*n* = 4				*n* = 20				*n* = 20				*n* = 8					
	♀	♂	Total	(%)	♀	♂	Total	(%)	♀	♂	Total	(%)	♀	♂	Total	(%)	♀	♂	Total	(%)	♀	♂	Total	(%)	Total (%)	
*Lutzomyia evansi*	427	56	483	84.0	366	440	806	88.5	189	243	432	76.1	41	20	61	66.3	104	90	194	65.5	243	133	376	65.3	2,352	77.9
*Lutzomyia panamensis*	10	0	10	1.7	43	27	70	7.7	70	29	99	17.4	4	0	4	4.3	24	8	32	10.8	92	71	163	28.3	378	12.5
*Lutzomyia gomezi*	13	2	15	2.6	18	4	22	2.4	17	9	26	4.6	2	1	3	3.3	11	4	15	5.1	6	5	11	1.9	92	3.0
*Lutzomyia dubitans*	14	6	20	3.5	1	3	4	0.4	1	1	2	0.4	5	2	7	7.6	13	9	22	7.4	3	5	8	1.4	63	2.1
*Lutzomyia trinidadensis*	21	5	26	4.5	3	0	3	0.3	2	0	2	0.4	7	0	7	7.6	5	1	6	2.0	7	3	10	1.7	54	1.8
*Lutzomyia cayennensis cayennensis*	7	10	17	3.0	2	1	3	0.3	2	0	2	0.4	2	8	10	10.9	2	7	9	3.0	1	2	3	0.5	44	1.5
*Lutzomyia rangeliana*	1	0	1	0.2	0	0	0	–	2	0	2	0.4	0	0	0	–	10	4	14	4.7	2	0	2	0.3	19	0.6
*Lutzomyia punctigeniculata*	3	0	3	0.5	0	0	0	–	0	0	0	–	0	0	0	–	3	0	3	1.0	2	1	3	0.5	9	0.3
*Lutzomyia shannoni*	0	0	0	–	0	1	1	0.1	2	1	3	0.5	0	0	0	–	1	0	1	0.3	0	0	0	–	5	0.2
*Lutzomyia camposi*	0	0	0	–	2	0	2	0.2	0	0	0	–	0	0	0	–	0	0	0	–	0	0	0	–	2	0.1
Total	496	79	575		435	476	911		285	283	568		61	31	92		173	123	296		356	220	576		3,018	

**Table 2:** Mean abundance by habitat of *Lutzomyia* species with remarkable vectorial importance, caught with CDC traps in Santa Cruz settlement (Atlántico, Colombia).

	Rural area						Peri-urban area					
	Indoors (n* = 8)		Peri-domestic area (*n* = 8)		Forest (*n* = 4)		Indoors (*n* = 20)		Peri-domestic area (*n* = 20)		Shrubland (*n* = 9)	
Phlebotomine sand flyspecies	GM_w_	CI 95%	GM_w_	CI 95%	GM_w_	CI 95%	GM_w_	CI 95%	GM_w_	CI 95%	GM_w_	CI 95%
*Lutzomyia evansi*	13.3	(1.7–72.3)	26.1	(3.5–162.7)	74.7	(15.5–345.6)	2.1	(1.2–3.4)	4.8	(2.3–9.0)	17.7	(4.8–59.5)
*Lutzomyia panamensis*	0.9	(0.1–2.2)	4.3	(0.8–14.7)	17.9	(3.2–82.8)	0.1	(0.006–0.3)	1.0	(0.5–1.8)	7.9	(1.8–27.6)
*Lutzomyia gomezi*	0.8	(−0.2–3.0)	2.3	(1.2–4.0)	6.2	(3.1–11.4)	0.1	(−0.01–0.2)	0.5	(0.2–0.9)	0.8	(0.1–1.9)

GMw = geometric mean of Williams of number of sand flies/CDC trap/night.

* Sample size.

### Indoors and peri-domestic areas.

In the rural area, *Lu. evansi* infested 100% (8/8) of the dwellings, with a GM_w_ of 13.3 sand flies/CDC trap/night. Of the other species with relative abundance ≤ 5%, *Lu. panamensis* and *Lu. gomezi* stand out owing to their vectorial importance, with a dwelling infestation rate of 62.5% (5/8) and 37.5% (3/8), respectively, although with a low mean abundance, < 1 sand fly/CDC trap/night (Table 2). In the peri-domestic area, the mean abundance of *Lu. evansi*, *Lu. panamensis*, and *Lu. gomezi* was greater than the abundance found indoors, although the differences were not significant.

In the peri-urban area, the *Lu. evansi* dwelling infestation was lower, 85% (17/20), than that in the rural area. Its mean abundance was also lower, that is, 2.1 and 4.8 sand flies/CDC trap/night in the indoors and peri-domestic areas, respectively, which are 6.3 times (Mann–Whitney U-test; *z* = −2.103, *P* = 0.035) and 5.4 times lower (without significant differences) than the abundance found in the same habitats in the rural area.

### Forest and shrubland.

In the rural area, particularly in the forest, the species with the highest relative abundance were *Lu. evansi*, *Lu. panamensis*, *and Lu. gomezi*. The mean abundances of these species in the forest were 5.6, 19.9, and 7.7 times higher than those obtained indoors, respectively, with the increase being statistically significant for *Lu. panamensis* (Mann–Whitney U test; *z* = 2.746, *P* = 0.006) and *Lu. gomezi* (Mann–Whitney U test; *z* = 1.952, *P* = 0.0509). The mean abundance of *Lu. evansi*, *Lu. panamensis*, and *Lu. gomezi* in the forest was also higher than the abundance found in the peri-domestic area but was only statistically significant for *Lu. gomezi* (Mann–Whitney U-test; *z* = 2.169, *P* = 0.0301) (Table 2).

In the peri-urban area, in the shrubland habitat, *Lu. evansi* and *Lu. panamensis* were the two most abundant species, with mean abundances that were 4.2 and 2.3 times lower than those recorded in the forest in the rural area, respectively, although the differences were not significant. Finally, the mean abundances of these two species in the shrubland habitat were significantly higher than those found indoors in the same peri-urban area (*Lu. evansi*: Mann–Whitney U-test; *z* = 2.922, *P* = 0.0035 and *Lu. panamensis*: Mann–Whitney U-test; *z* = 3.573, *P* = 0.0004) (Table 2).

### Sampling with Shannon traps.

A total of 597 sand flies were sampled using Shannon traps. In the rural areas, 183 sand flies were collected in the peri-domestic area (*n* = 1): 90.7% were *Lu. evansi*, 6% were *Lu. panamensis*, 2.7% were *Lu. gomezi*, and 0.5% were *Lu. c. cayennensis*. In the forest habitat (*n* = 1) in rural areas, 369 sand flies were collected, corresponding to the following species: *Lu. evansi* (94.8%), *Lu. panamensis* (2.7%), *Lu. gomezi* (1.3%), *Lu. shannoni* (0.8%), and *Lu. c. cayennensis* (0.3%). Finally, in the peri-urban area, 45 sand flies were collected in the shrubland habitat: 64% were *Lu. evansi*, 28.9% were *Lu. gomezi*, and 6.7% were *Lu. c. cayennensis*.

### Human-landing catches of sand flies.

A total of 281 sand flies were sampled using protected human bait. The most abundant species was *Lu. evansi* (74.4%), followed by *Lu. gomezi* (12.1%) and *Lu. panamensis* (8.5%).

**Figure 2.**
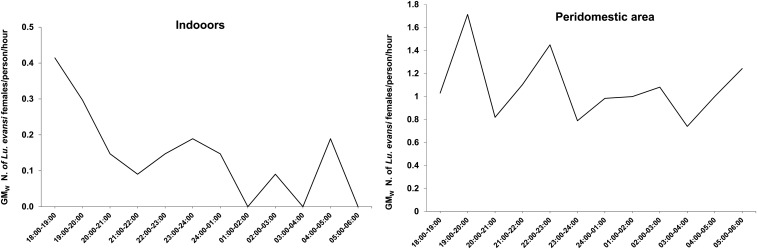
Activity of *Lutzomyia evansi* from 18:00 to 6:00 hours (females landing on human bait/hour) in the indoors and peri-domestic areas in the rural area of the Santa Cruz settlement (*n* = 4). In each dwelling, sampling was conducted for two consecutive nights.

Figure 2 shows the activity of *Lu. evansi* females from 18:00 to 6:00 hours. The activity of *Lu. evansi* was low within dwellings compared with than in the peri-domestic area (32 versus 169 females). The maximum human-landing activity indoors was observed at 18:00–19:00 hours and 19:00–20:00 hours, with GM_w_ values of 0.4 and 0.3 females/person/hour, respectively, followed by low but continuous activity overnight and in the early morning. In the peri-domestic area, females landed on the human bait throughout the sampling period, with the activity peaking between 19:00 and 20:00 hours (GM_w,_ 1.7 females/person/hour) and between 22:00 and 23:00 hours (GM_w,_ 1.4 females/person/hour). Some *Lu. evansi* males were also collected, eight in total, three in indoors and five in the peridomestic area, and were distributed throughout the sampling period.

Regarding *Lu. gomezi*, 33 females were sampled in total, six indoors (two females from 18:00 to 19:00 hours and four from 1:00 to 4:00 hours) and the other 27 were caught in the peri-domestic area (half of them between 19:00 and 20:00 hours and others distributed throughout the early morning). One *Lu. gomezi* male was collected in the peri-domestic area at 2:00 hours. In relation to *Lu. panamensis*, a total of 24 females were collected, one indoors at midnight and 23 in the peri-domestic area, nine of which were collected between 18:00 and 22:00 hours and 14 between midnight and 04:00 hours.

Some specimens of *Lu. trinidadensis* (total = 7), *Lu. c. cayennensis* (total = 5), and *Lu. shannoni* (total = 2) were also collected. For the first species, three females and one male were collected indoors between 21:00 and 2:00 hours, and three females were collected in the peri-domestic area, one at 18:00 hours and the other two before 4:00 hours. For the second species, the females were collected after midnight, two indoors and three in the peri-domestic area. Finally, one female of *Lu. shannoni* was collected indoors, and one in the peri-domestic area, both between 19:00 and 20:00 hours.

### Diurnal resting site catches of sand flies.

In the eight dwellings inspected, a total of 52 sand flies were sampled by direct aspiration at resting sites. Table 3 shows the species composition, the specific collection sites, the trophic condition of the females, and the presence of mature eggs. The most abundant species was *Lu. c. cayennensis* (44.2%), followed by *Lu. trinidadensis* (26.9%) and *Lu. evansi* (21.2%); these species were found in approximately half of the dwellings. The other two species observed at very low abundances (< 6.0%) were *Lu. shannoni* and *Lu. panamensis*, both with a dwelling infestation percentage of only 12.5%.

**Table 3:** *Lutzomyia* spp. collected by direct aspiration from diurnal resting sites in rural dwellings (*n* = 8) from Santa Cruz settlement (Atlántico, Colombia).

Phlebotomine sand flies species	♀	♂	Total	%	Positive dwellings (%)	Phlebotomine sand flies found by collection site % (No. ♀, No. ♂)				Females trophic status and mature eggs presence (%)				
						Indoors	Peri-domestic area							
						Bedrooms	Bathroom	Chicken roost	Tree trunks	Without blood or mature eggs	Fresh blood	Digested blood	Digested blood and mature eggs	Mature eggs
*Lutzomyia cayennensis*	14	9	23	44.2	50.0	82.6 (13♀, 6♂)	0	17.4 (1♀, 3♂)	0	42.9	28.6	7.1	14.3	7.1
*Lutzomyia trinidadensis*	14	0	14	26.9	50.0	64.3 (9♀, 0♂)	0	7.1 (1♀, 0♂)	28.6 (4♀, 0♂)	50.0	35.7	7.1	0	7.1
*Lutzomyia evansi*	8	3	11	21.2	62.5	91.0 (7♀, 3♂)	9.0 (1♀, 0♂)	0	0	37.5	37.5	25.0	0	0
*Lutzomyia shannoni*	1	2	3	5.8	12.5	0	0	0	100 (1♀, 2♂)	0	100	0	0	0
*Lutzomyia panamensis*	0	1	1	1.9	12.5	0	100 (1♀, 0♂)	0	0	0	0	0	0	0
Total	37	15	52											

Regarding the specific sites within the dwelling where sand flies were found resting, for the three most frequent species, the bedroom walls were the place where a higher percentage of these insects were found.

In general, a greater number of females were sampled for most species. With regard to the female trophic status, in the three most abundant species, approximately one-third of the females contained red fresh blood (28.6–37.5%), whereas a low percentage had digested blood (7.1–25.0%). Finally, taking the gravid stage into account, four females had mature eggs, one *Lu. trinidadensis* and three *Lu. c. cayennensis*; of the latter three, two females also contained digested blood.

## DISCUSSION

Considering the political-administrative division of Colombia, the Atlántico department borders endemic areas of CL and VL and is also located in the same Holdridge life zones where there have been outbreaks of the disease in neighboring departments (dry and very dry tropical forests). Therefore, the presence of insect vectors of *Leishmania* and reports of autochthonous cases of leishmaniasis in the department were expected. Local entomological characterization and its association with the results of foci studies on the disease in the Caribbean region of Colombia allowed us to define the species of sand flies that could be considered vectors in the area, with the purpose focusing on continuous entomological surveillance of these species.

In several leishmaniasis foci in the Caribbean region of Colombia, the composition of sand fly species is similar, with *Lu. evansi* being predominant over *Lu. panamensis* and *Lu. gomezi.* Other species that have generally been found are *Lu. trinidadensis*, *Lu. c. cayennensis*, *Lu. rangeliana*, *Lu. dubitans*, and *Lutzomyia walkeri*.^7,8^ Some considerations regarding the importance of these species in public health in comparison with the findings of studies conducted in the Caribbean region of Colombia have been set out in the following text.

*Lutzomyia evansi* was the predominant species both in rural and peri-urban areas, with relative abundances of > 76% and > 65%, respectively, and infested 100% and 85% of the dwellings sampled in each area. The high abundance of *Lu. evansi* coincides with the findings of previous studies on leishmaniasis foci in the departments of Sucre,^8–10^ Bolívar,^7,11^ and Córdoba,^12^ where the relative abundance of this species fluctuated between 70% and 90% and where *Lu. evansi* was considered the most likely vector in each focus. The predominance of a single resistant species could be associated with both the loss of a large proportion of the original habitat and severe habitat fragmentation,^13^ which are both features of the tropical dry forest of northern Colombia because of agricultural activities.^14^

In the absence of *Lutzomyia longipalpis*, *Lu. evansi* is the main vector of the parasite *Leishmania infantum*, responsible for the occurrence of VL,^15^ and natural infection of *Lu. evansi* with *L. infantum* has been reported in different localities of the Caribbean region of Colombia.^12,16^ In addition, studies conducted particularly in the Montes de María region suggest that *Lu. evansi* could be involved in transmitting the etiological agent of CL because this sand fly species was found naturally infected with flagellates of *Leishmania braziliensis* and because nucleotide sequence analysis allowed this parasite to be grouped with isolates of *L. braziliensis* from patients with CL residing in the same area.^17^

*Lutzomyia panamensis* and *Lu. gomezi* are two other species found in both rural and peri-urban areas. These species are anthropophilic and have been implicated as suspected vectors in the CL transmission cycle in several foci in the Caribbean region of Colombia, particularly in the departments of Córdoba,^12,18^ Sucre,^8^ and Bolívar.^11^ They have also been associated with the transmission of *Leishmania panamensis* and *L. braziliensis* in different CL foci in Central and South America.^19,20^

In addition, we recorded species such as *Lu. c. cayennensis*, which has been found to be naturally infected with trypanosomatids in Montes de María in the Caribbean region of Colombia,^21^ as well as *Lu. trinidadensis* and *Lu. rangeliana* which in Venezuela were found with parasites of *Leishmania venezuelensis* and *Leishmania* spp., respectively.^22,23^ However, the role of these species in the transmission of CL in the Caribbean region of Colombia remains to be determined.

The presence of the three proven or probable vectors *Lu. evansi*, *Lu. panamensis*, and *Lu. gomezi* in different habitats (indoors, peri-domestic area, forests, and shrublands) and sampled areas (rural and peri-urban areas) shows the versatility and dispersal capacity of these species and their adaptation to highly human-disrupted habitats.^8,24^ However, habitat types sampled outside the dwelling are rarely mentioned in *Leishmania* spp. foci studies in the Caribbean region of Colombia. Studies include outdoors areas, but without defining the type of habitat sampled.^7,25^ In the present study, the outdoors consisted two types of habitats, forest in the rural area and shrubland in the peri-urban area; the latter type of habitat is very common in the tropical dry forest life zone in the Caribbean region of Colombia,^14^ but had not been previously sampled. Abundances of the three probable vectors were higher in forest and shrubland than in indoor and peri-domestic habitats in each area and were higher in the forest than in shrubland.

The aforementioned observations coincide with the findings of a study that examined changes in the phlebotomine fauna resulting from human intervention in a tropical dry forest of northern Colombia,^10^ which found that habitat degradation negatively affected the abundance and diversity of phlebotomine populations, but that medically important species, including *Lu. evansi*, *Lu. panamensis*, and *Lu. gomezi*, were able to exploit modified environments, maintaining a risk factor for the transmission of *Leishmania*.

On the other hand, for the three probable vectors of *Leishmania* spp., *Lu. evansi*, *Lu. panamensis*, and *Lu. gomezi*, this study presents an indicator that has been overlooked in other studies carried out in the Caribbean region of Colombia, that is, the abundance of the species in each habitat, expressed as the geometric mean of the number of sand flies/sampling method/night. This is an indicator of great importance that can be used to estimate the level of nuisance caused by a hematophagous insect or to design studies to identify the determinants that condition these abundances. In addition, this metric can be used in the evaluation of the efficacy of vector control interventions as an entomological indicator that allows comparison of the impact of these measures.^26^

The CL case that motivated this study could be considered autochthonous from Luruaco in the department of Atlántico for two reasons: 1) the 65-year-old man with a positive diagnosis of CL reported not having traveled outside the municipality for at least 1 year before the lesion appeared and 2) it was confirmed that in the sites of possible transmission, that is, rural and peri-urban areas of the municipality of Luruaco, there are ecological conditions that facilitate the proliferation of vector sand flies. As of 2009, new cases of CL have not been notified in the department of Atlántico by SIVIGILA; however, it is not ruled out that new cases have occurred that have not been submitted to the health system because it is considered that the under-registration for CL remains high in many regions.^27,28^

The single case report of CL could be explained by the zoonotic nature of the disease, where transmission to a human depends on his or her contact with the vector in biomes where the enzootic cycle exists.^29^ In the study area, it is likely that the transmission cycle of *L. braziliensis* or *L. panamensis*^30^ has been maintained, over time, at an enzootic level involving wild mammals and sand flies, both with a low level of interaction with humans. However, in recent decades, as has happened in other areas, the loss of mammalian biodiversity as a result of deforestation and agricultural practices may have forced vectors to feed on humans and on a smaller number of synanthropic reservoirs.^31^

On the other hand, it should be considered that the delimitation of the foci of leishmaniasis is not because of political-administrative divisions but mainly to the distribution of reservoirs and vectors; therefore, the area of transmission of *Leishmania* spp. causing the CL may include, among others, municipalities in the departments of Bolivar, Atlántico, and Magdalena. As an example of the aforementioned, in 2009, of the cases reported in the neighboring department of Bolivar, two had the municipality of Villanueva as the origin (SIVIGILA), which is located to the southeast of the municipality of Luruaco. However, political-administrative divisions are relevant to plan at the national level regarding the allocation of resources for the prevention and control of leishmaniasis.

### Behavioral aspects of phlebotomine sand flies in the study area.

Regarding the human-landing activity of sand flies, from the 10 species recorded by caught using CDC and Shannon traps, three species mainly approached the volunteers to feed, *Lu. evansi*, *Lu. gomezi*, and *Lu. panamensis*, thereby confirming the anthropophilic behavior of these species in the sampled area of the Atlántico department.

*Lutzomyia evansi* landed on the volunteers at a higher abundance; hence, it was possible to establish an activity pattern for this species. Inside the houses, the peak activity was observed from 18:00 to 20:00 hours, with a low but continuous activity overnight and in the early morning. In the peri-domestic area, sustained activity took place during the night, although with two peaks between 19:00 and 23:00 hours. This observation partly coincides with a study about the nocturnal human-landing activity of *Lu. evansi* inside a house in Anzoátegui State, Venezuela, where the species was also mostly active at night (from 19:00 to 5:00 hours), although the maximum activity occurred near midnight (from 23:00 to 00:00 hours).^24^ In the Caribbean region of Colombia, particularly in San Andrés de Sotavento, Córdoba, collections performed using protected human bait in the peri-domestic habitat throughout the night indicated that most sand flies were active late at night and during the early morning hours (from 00:00 to 3:00 hours), but the sand flies species were not identified, representing a weakness of that study.^16^

Our research reports, for the first time in the Caribbean region of Colombia, result about the following of sand fly human-landing activity throughout the night (18:00 to 6:00 hours), discerning this activity for each *Lutzomyia* species.

Regarding the endophilic resting behavior, entomological studies on CL and VL foci in the Caribbean region of Colombia have shown that phlebotomine sand flies use the roots and trunks of different tree species both around the houses and outdoors as diurnal resting places.^25,32^ During active searches of sand flies in these microhabitats in the Córdoba department, *Lu. panamensis*, *Lu. gomezi*, *Lu. shannoni*, and *Lu. trinidadensis* were caught,^18^ and in the Sucre department, 11–15 species of sand flies were collected, with *Lutzomyia micropyga*, *Lu. evansi*, and *Lu. trinidadensis* being the most abundant.^32,33^

Outside the dwellings, the findings of *Lu. shannoni* and *Lu. trinidadensis* resting in tree trunks in the Santa Cruz settlement coincide with the species recorded in previous studies.^32,33^

Our results constitute the first report or quantification of phlebotomine sand flies resting in indoor sites in the Caribbean region of Colombia. Of the total sand flies captured through active searches at potential resting sites, 73.1% were collected from bedroom walls, and between 7.1% and 25% of the females collected had digested blood and/or mature eggs. Therefore, the possibility that the bedroom wall is one of the surfaces on which females rest after blood feeding, carrying out part of the blood digestion process and the development of eggs, could not be ruled out. The aforementioned possibility is relevant considering that, in the definition of intradomiciliary control measures, it is important to establish indicators related to the endophilic degree for possible vectors to consider or discard measures such as indoor spraying with residual insecticides.^34^

### Contribution of the study to the entomological surveillance and prevention of CL.

Because of their vectorial importance, the species that could be considered probable vectors of leishmaniasis in the Santa Cruz settlement, Luruaco municipality, and on which entomological surveillance should be focused are as follows: *Lu. evansi*, *Lu. panamensis*, and *Lu. gomezi*. The risk of human–vector contact was observed both in the rural and peri-urban areas sampled, both inside and outside dwellings. *Lutzomyia evansi* was the predominant species, and its endophagy was confirmed by human-landing catches inside the houses throughout the night. Regarding the endophilic behavior, bedroom walls were the resting sites for this species.

An objective of vector control in leishmaniasis is to reduce the exposure of people to phlebotomine sand fly bites, which is partly achieved by prevention measures that reduce human–vector contact.^35^ The endophagy of *Lu. evansi* and its human-landing activity inside dwellings coincide with the hours during which the inhabitants are sleeping, indicating that bed nets treated with pyrethroids could be helpful. However, considering the warm climate of the study area and the community acceptability, an appropriate mesh size that allows for adequate ventilation and the use of an insecticide (at an adequate dose) that decreases sand fly passage through bed nets and/or has a lethal effect on them should be established.^34^

In addition to the inside of dwellings as a risk site for human–vector contact, the peri-domestic area where *Lu. evansi* proved to be particularly active from 18:00 to 21:00 hours was also considered important. Sampling with CDC and Shannon traps also demonstrated the presence of the three probable vector species in outdoor settings, such as forests and shrublands. Regarding the outdoor settings, the suggested protection measures against sand fly bites are using clothing that covers the arms and legs and/or using topical repellents.^36^

Finally, considering that the confirmed case of CL in the study area was an isolated event, it could not justify a large-scale vector control program. Epidemiological surveillance should be strengthened to allow for detection, diagnosis, and opportune treatment of new cases of CL in the Atlántico department. In addition, educational strategies directed toward the community should be adopted to address the disease symptoms, and insect vector identification and prevention measures that can help reduce human–vector contact should be adopted.

## CONCLUSION

The entomological characterization reported in the present study can be useful as a baseline for both requirements, that is, for conducting detailed studies on possible vectors (e.g., seasonal variation and natural infection with *Leishmania* spp.) or for implementing continuous entomological surveillance in the area, to monitor changes in the abundances or in the behavior of probable vectors and to allow for the proposal of adequate and timely entomological management when possible outbreaks of leishmaniasis happen.
